# The complete plastid genome of *Zenia insignis* Chun (Leguminosae)

**DOI:** 10.1080/23802359.2019.1660266

**Published:** 2019-09-05

**Authors:** Qiang Lai, Tieyao Tu, Dianxiang Zhang

**Affiliations:** a Key Laboratory of Plant Resources Conservation and Sustainable Utilization, South China Botanical Garden, Chinese Academy of Sciences, Guangzhou, China;; b University of Chinese Academy of Sciences, Beijing, China

**Keywords:** Dialioideae, threatened species, legume family, chloroplast genome, phylogeny

## Abstract

*Zenia insignis* Chun is a rare and threatened species of the monotypic genus *Zenia* Chun in the subfamily Dialioideae of Leguminosae. The complete plastid genome of *Zenia insignis* was sequenced for the first time which also represents the first complete plastid genome of the subfamily Dialioideae. The total length of this genome is 159,358 bp with a large single copy (LSC) region (88,507 bp), a small single copy (SSC) region (18,689 bp), and two inverted repeat regions (IRs, 26,081 bp). We recovered 128 functional genes, including 83 protein-coding genes, 37 tRNA genes and 8 rRNA genes. Phylogenetic analysis strongly supported a sister relationship between *Z. insignis* and the clade consisting of Caesalpinioideae and Papilionoideae.

*Zenia insignis* Chun, a rare and threatened tree species of the subfamily Dialioideae of the legume family, is the only species of the genus *Zenia* Chun (LPWG 2017), occurring in limestone habitats in southern China and northern Vietnam (Chun 1946; Chen et al. 2010). It is listed in the Plant Red Data Books of China under second-class national protection (Fu and Jin 1992) and also in the IUCN Red List (IUCN 2019) under the VU rating. Here we firstly reported the complete plastid genome of *Z. insignis* which also represents the first complete plastid genome or chloroplast genome of Dialioideae. Knowledge on the genome of *Z. insignis* will be useful for studying the conservation of this species and for recovering its phylogenetic position within the legume family.

The total genomic DNA was isolated using fresh and healthy leaf tissues with a modified CTAB method (Doyle and Doyle 1987). The voucher specimen (LaiQ033) was collected from South China Botanical Garden, Guangzhou, China (113°21′54.07″E, 23°10′59.30″N) and deposited in the herbarium of South China Botanical Garden (IBSC). Paired-end (PE) sequencing was conducted on the Illumina HiSeq X-Ten instrument at Beijing Genomics Institute (BGI) in Wuhan, China. We employed GetOrganelle pipeline (Jin et al. 2018) and PGA program (Qu et al. 2019) to assemble and annotate the plastome. Geneious v.9.1.8 (Kearse et al. 2012) was used to verify the accuracy of the assembly, and to correct the start/stop codons and intron/exon boundaries of the annotation. The annotated plastome was deposited in GenBank (accession number: MN116508).

To reconstruct the phylogenetic position of *Z. insignis*, we combined our newly generated data with nine previously published plastid genomes which represent the outgroup of Quillajaceae and all the subfamilies of Leguminosae except for Duparquetioideae (LPWG 2017) (Figure 1). We aligned 77 protein-coding genes using MAFFT V.7.308 (Katoh and Standley 2013) with default parameters and manually adjusted misaligned regions using Geneious. We reconstructed the maximum likelihood phylogeny based on a concatenation of the 77 genes using RA x ML v.8.2.10 (Stamatakis 2014) with the GTR + G substitution model as suggested by ModelFinder (Kalyaanamoorthy et al. 2017).

**Figure 1.**
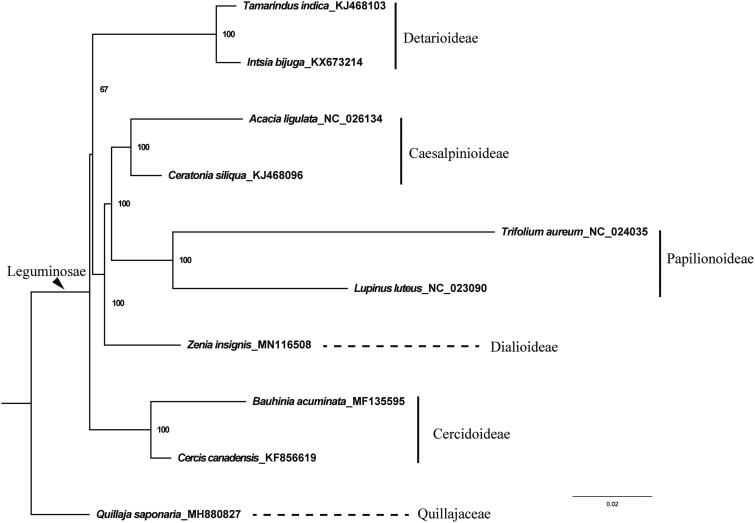
The maximum-likelihood (ML) phylogenetic tree based on the concatenation of 77 protein-coding sequences. Numbers at the right of nodes are bootstrap support values.

The complete plastid genome of *Zenia insignis* was 159,358 bp in length with a typical quadripartite organization: a large single copy (LSC) region of 88,507 bp, a small single copy (SSC) region of 18,689 bp, and two inverted repeat regions (IRa and IRb), each of 26,081 bp. A total of 128 functional genes were recovered, consisting of 83 protein-coding genes, 37 tRNA genes and 8 rRNA genes. The overall GC content of the whole plastome is 35.3%.

Our phylogenetic analysis strongly suggested that *Zenia insignis* (Dialioideae) is sister to a clade composed by Caesalpinioideae and Papilionoideae with a strong bootstrap support. We recovered Cercidoideae as the basalmost lineage in the family and the grouping of the other four subfamily received a moderate bootstrap support (Figure 1). Our study suggested potential benefit of complete plastid genome in inferring the phylogenetic relationships within the legume family.
